# Functional Characterization of TLR4 +3725 G/C Polymorphism and Association with Protection against Overweight

**DOI:** 10.1371/journal.pone.0050992

**Published:** 2012-12-11

**Authors:** Alberto Penas-Steinhardt, Lucía Soledad Barcos, Fiorella Sabrina Belforte, Martha de Sereday, Jorge Vilariño, Claudio Daniel Gonzalez, María Teresa Martínez-Larrad, Mariana Lorena Tellechea, Manuel Serrano-Ríos, Edgardo Poskus, Gustavo Daniel Frechtel, Federico Coluccio Leskow

**Affiliations:** 1 Instituto de Estudios de la Inmunidad Humoral (IDEHU), CONICET-UBA y Cátedra de Inmunología - Facultad de Farmacia y Bioquímica, Universidad de Buenos Aires, Ciudad Autónoma de Buenos Aires, Buenos Aires, Argentina; 2 División Endocrinología, “Hospital de Clínicas”, Universidad de Buenos Aires (UBA), Ciudad Autónoma de Buenos Aires, Buenos Aires, Argentina; 3 Departamento de Química Biológica, Facultad de Ciencias Exactas y Naturales, Universidad de Buenos Aires, IQUIBICEN-CONICET, Ciudad Autónoma de Buenos Aires; & Departamento de Ciencias Básicas, Universidad Nacional de Lujan, Luján, Buenos Aires, Argentina; 4 Laboratorio de Biología Molecular, Cátedra de Genética y Biología Molecular, Facultad de Farmacia y Bioquímica, Universidad de Buenos Aires, Ciudad Autónoma de Buenos Aires, Buenos Aires, Argentina; 5 Servicio de Endocrinología, Hospital Fiorito, Buenos Aires, Argentina; 6 Cardiología Clínica, Instituto FLENI, Ciudad Autónoma de Buenos Aires, Buenos Aires, Argentina; 7 Departmento de Farmacología, Facultad de Medicina, Universidad de Buenos Aires, Buenos Aires, Argentina; 8 Spanish Biomedical Research Centre in Diabetes and Associated Metabolic Disorders (CIBERDEM), Instituto de Investigación Sanitaria del Hospital Clínico San Carlos (IdISSC), Madrid, Spain; 9 División Genética, “Hospital de Clínicas”, Universidad de Buenos Aires (UBA), Ciudad Autónoma de Buenos Aires, Buenos Aires, Argentina; University of Siena, Italy

## Abstract

Subclinical low-grade systemic inflammation has been associated with obesity, insulin resistance and metabolic syndrome (MS). Recent studies have highlighted the role of gut microbiota in these disorders. The toll-like receptor 4 (TLR4) plays a key role in the innate immune response activation. We studied two polymorphisms (+3725G/C and 11350G/C) in the 3′ untranslated region (3′UTR) of the TLR4 gene that may alter its expression and their association with metabolic disorders related to systemic inflammation. We cloned the 3′UTR into a luciferase reporter system and compared wild-type 3′UTR (WT) and +3725C variant (MUT) constructs luciferase activities. MUT construct reduced the reporter gene activity by 30% compared to WT (P = 0.0001). To evaluate the association between these polymorphisms with biochemical and clinical overweight related variables, we conducted a population cross-sectional study in 966 men of Argentine general population. Considering smoking as a confounding variable that causes systemic inflammation, we studied these possible effects in both, smokers and nonsmokers. The 11350G/C polymorphism was not detected in our sample whereas the CC genotype of +3725 polymorphism was associated with lean subjects (p = 0.011) and higher Adiponectin levels (p = 0.021). Subjects without any NCEP/ATP III MS component were associated with this genotype as well (p = 0.001). These results were strengthened in nonsmokers, in which CC genotype was associated with lean subjects (p = 0.003) and compared with G carriers showed significantly lower BMI (25.53 vs. 28.60 kg/m2; p = 0.023) and waist circumference (89.27 vs. 97.51 cm; p = 0.025). None of these associations were found in smokers. These results showed that +3725C variant has a functional effect down-regulating gene expression and it could be considered as a predictive factor against overweight, particularly in nonsmokers. Considering the role of TLR4 in inflammation, these findings would suggest that the presence of +3725C variant could predict a lower prevalence of chronic metabolic disorders.

## Introduction

Obesity and Type 2 Diabetes Mellitus (T2DM) prevalence has increased throughout the world, mainly due to rapid lifestyle changes [1]. Since the early 60’s James Neel proposed the “thrifty genotype hypothesis” explaining the significant increase of diabetes already observed at that time [2]. However, it is currently believed that this increase cannot be attributed solely to changes in nutritional habits or reduction of physical activity in daily life [3].

Subclinical low-grade systemic inflammation has been associated with obesity, metabolic syndrome (MS) and T2DM [4]. Several studies in animal models as well as in humans have shown that changes in the innate immune response are involved in the pathogenesis of these metabolic disorders [5]. Recent studies have highlighted the role of the gut microbiota in the perpetuation of both insulin resistance (IR) and low-grade chronic inflammation [6], suggesting an alternative explanation for the increasing of IR related diseases prevalence. The gut microbiota, whose collective genome has been estimated to contain 100 times more genes than the human genome [7], is in permanent contact with nutrients and interacting with the host’s cells. The composition of this complex ecosystem varies according to the quality and quantity of food [8]. This provides a direct relationship between development of the MS, dysfunction of the innate immune system, and changes in the composition of the gut microbiota [9].

The adipose tissue is an important endocrine and secretory organ, which releases a wide range of signals and specific factors termed adipokines, such as Leptin and Adiponectin [10]–[11]. These bioactive molecules play a pivotal role in metabolism and energy homeostasis. Adiponectin deficiency results in insulin resistance, glucose intolerance and hypertriglyceridemia. Epidemiological studies have shown that low levels of Adiponectin predicted later development of T2DM [12].

On the other hand, as confirmed by numerous population-based studies, a low-grade systemic inflammatory response is also evident in smokers: elevated levels of C-reactive protein (CRP), fibrinogen, and interleukin-6, as well as increased counts of white blood cells (WBC) have been reported [13].

In Drosophila, Toll mediates dorso-ventral embryonic polarization and activates immune responses to fungal infection. A human homologue of the fly [14], the toll-like receptor 4 (TLR4), is a leucine-rich repeat molecule that plays a key role in the activation of innate immune response by recognizing conserved pathogen-associated molecular patterns, such as lipopolysaccharides (LPS) [15]. The activation of TLR4 signaling induces up-regulation of inflammatory pathways related to the induction of IR. It was found that in C3H/HeJ mice a loss-of-function mutation (Pro^712^His) in TLR4 prevents diet-induced obesity and IR [16].

Additionally, two miss-sense mutations in TLR4 gene (Asp299Gly and Thr399Ile) have been reported to be associated with endotoxin hyporesponsivenes [17]. Further genetic association studies have leaded to contradictions regarding the role of both polymorphisms and their possible effects on inflammatory and metabolic disorders like atherosclerosis, IR, MS and DM2 [18]–[19].

The control of mRNA stability is an important process that allows cells to rapidly adjust protein levels providing a fine tuning mechanism to regulate gene expression. Variations in the 3′-UTR are known to altered gene expression levels through a direct effect on mRNA stability or translation efficiency. In particular, two polymorphisms +3725G/C (rs11536889) and 11350G/C (rs41426344) in the 3′UTR of TLR4 gene were recently found to be associated with alterations in the immune response [20]–[21].

Taking into account the role of TLR4 as a molecular gate linking inflammation with insulin resistance, diabetes, and obesity [6] and that genetic associations may vary largely with environmental factors [22], we propose to study if the smoking habit influences the effect of genetic variation.

In this study we present the analysis of the functional effect of +3725G/C and 11350G/C sequence variants of TLR4 gene on mRNA stability and the association between these polymorphisms and markers of inflammation, MS and metabolic associated traits in non diabetic men, focusing especially on the potential interaction with smoking status.

## Materials/Subjects and Methods

### Ethics Statement

Each subject signed a consent form to participate in this genetic study. The study was carried out in accordance with the Declaration of Helsinki, and approved by the Ethic and Research committees of the “Hospital de Clínicas”, Universidad de Buenos Aires (UBA), Argentina.

### Subjects

All 966 male individuals were unrelated subjects from general Argentinian population self-reporting European ancestry, especially from southern European countries (Spain and Italy), living in Buenos Aires metropolitan area, recruited between April 2006 and April 2010. The clinical characteristics of the sample are shown in Table 1.

**Table 1:** Clinical characteristics for all participants, stratified by TLR4+3725G/C (rs11536889) genotype and smoking status.

	All subjects	TLR4+3725G/C			Smoking status		
	n = 966	G/G+G/C (n = 949)	C/C (n = 17)	p	non smoker (n = 585)	Smoker (n = 381)	p
	Mean±SD	Mean±SD	Mean±SD		Mean±SD	Mean±SD	
Age (yr)	39.67±12.85	39.71±12.83	37.41±13.36	ns	40.09±13.51	38.95±11.843	ns
BMI (kg/m^2^)	28.42±4.79	28.45±4.80	26.41±4.16	ns	28.54±4.62	28.18±5.049	ns
WC (cm)	97.07±13.04	97.15±12.97	91.20±13.28	ns	97.33±12.75	96.55±13.53	ns
SBP (mmHg)	127.06±14.52	127.12±14.57	121.00±11.75	ns	127.50±13.91	126.33±15.46	ns
DBP (mmHg)	80.06±9.35	80.09±9.36	76.88±8.73	ns	80.12±8.94	79.86±9.96	ns
TC (mg/dl)	187.42±39.70	187.66±39.89	180.00±36.35	ns	187.27±38.96	187.72±40.95	ns
LDL-C (mg/dl)	117.25±34.37	117.32±34.47	115.32±31.23	ns	117.52±33.33	116.86±35.92	ns
HDL-C (mg/dl)	42.17±10.82	42.08±10.79	44.76±9.01	ns	42.62±10.76	41.61±10.89	ns
TG (mg/dl)	139.95±96.31	141.24±97.44	99.53±47.28	ns	135.57±93.50	146.11±100.45	ns
FPG (mg/dl)	94.56±21.67	94.68±21.70	91.82±7.57	ns	95.10±20.07	93.86±24.07	ns
hsCRP (mg/L)	1.96±1.88	1.97±1.89	1.32±1.22	ns	1.85±1.87	2.11±1.88	0.010*
Adiponectin^a^ (ng/ml)	10.68±4.35	10.64±4.34	14.48±5.62	0.021*	10.55±4.32	10.96±4.40	ns
Fasting insulin^b^	16.25±9.31	13.48±4.96	16.30±9.37	ns	16.18±9.14	16.16±9.47	ns
HOMA-IR	2.07±1.17	1.74±0.64	2.08±1.17	ns	2.07±1.15	2.06±1.18	ns

a Adiponectin was measured in 467 individuals.

b Fasting insulin was measured in 730 individuals.

Differences in the continuous traits values among the genotype groups were analyzed by ANOVA (log variable transformation was performed when variables showed non normal distribution). p<0.05 is indicated by *.

BMI, body mass index; WC, waist circumference; SBP, systolic blood pressure; DBP, diastolic blood pressure; TC, total cholesterol; LDL-C, low-density lipoprotein cholesterol; HDL-C, high-density lipoprotein cholesterol; TG, triglycerides; FPG, fasting plasma glucose; HOMA-IR, homeostasis model assessment of insulin resistance.

### Bioinformatic Analysis

Online programs UTRSCAN (www.ba.itb.cnr.it/BIG/UTRScan/) and Targetscan (http://www.targetscan.org/) were used for searching putative UTR functional elements [23]. The RNA secondary structure was predicted by online program Mfold version 3.1 (www.bioinfo.rpi.edu/applications/mfold/) [24]. Comparative genomic alignment was performed by MULTIZ algorithm (Human Gene Sorter, University of California, Santa Cruz (UCSC)) [25].

### 3′ UTR Luciferase Reporters

Genomic DNA from peripheral blood from homozygous individuals for each variant was used as template in a polymerase chain reaction (PCR). The entire TLR4 3′UTR (2908 bp) was amplified using the primers: TLR4_3′UTR_F: 5′-GCGCTAGCAGGAAGCAACATCTATCTGAAGAGGAAAAA-3′ and TLR4_3′UTR_R: 5′-GCATCGATTGGTCAGTTATGGTCTGAAAATAGGTGAGT-3′. Wild type (WT) and mutant TLR4 +3725C (MUT) constructs were obtained by cloning the PCR products into the pCR2.1 vector by using a TOPO-TA cloning kit (Invitrogen). Plasmid DNA was prepared using the Qiagen plasmid Maxi kit (Qiagen, Hilden, Germany). The primers contain two recognition sites for enzymes NheI and ClaI respectively (underlined above) to enable cloning into the firefly luciferase expression plasmid (pTRE-Luc, Clontech Laboratories, Inc.). Luciferase constructs were verified by sequencing.

### Cell Culture and Transfections

HeLa Tet-off advanced (Clontech) cells were manteined in DMEM High Glucose medium (Invitrogen, CA, USA) supplemented with 10% fetal bovine serum (FBS), 100 units/ml Potassium Penicillin, 100 µg/ml Streptomycin Sulfate, 250 ng/ml Amphotericin B at 37°C in a humidified chamber containing 5% CO2. Cells (200.000 cells/well) were seeded in a 12-well plate 24 h before transfections. Each construct (1 µg) was co-transfected with pCMV β-gal plasmid (100 ng) as an internal control using 3 µl of Polyethylenimine (PEI) 1 µg/ml. The pCMV β-gal plasmid contains the reporter gene β-galactosidase (β-gal) from *E. coli*. Twenty four hours after transfection, the cells were harvested and assayed for firefly luciferase and β-gal activities using Luciferase Assay Reagent (Promega) and ortho-Nitrophenyl-β-galactoside (ONPG) respectively. Measurement of luciferase activity was performed on the GloMax®-Multi Detection System Promega. The relative firefly luciferase activities were normalized against the β-gal activities.

### Biochemical Analyses, Anthropometric and Clinical Measurements

Anthropometric measurements (height, weight, body mass index and waist circumference), systolic and diastolic blood pressure (SBP and DBP, respectively) were determined by a standardized protocol. After a 12-h overnight fast, blood samples (20 ml) were drawn in every individual, centrifuged and frozen immediately at –20°C. Total cholesterol (TC), triglycerides (TG), and high-density lipoprotein cholesterol (HDL-C) were determined in serum by enzymatic methods using commercial kits (TG Triglycerides GPO-PAP, CHOL Cholesterol CHOD-PAP and Phosphotungstate Precipitant, Roche Diagnostics, Mannheim, Germany) in a Hitachi 727 autoanalyzer. All determinations were performed using standardized procedures. Intra-CV (coefficient of variation) for TG and total cholesterol were 1.3 and 1.1%, respectively. Inter-CVs were 2.4% and 1.5%, respectively. LDL-C (low-density lipoprotein cholesterol) was calculated using the Friedewald formula (LDL-C = TC – HDL-C – [TG/5].

Fasting plasma glucose (FPG) was determined by a glucose-oxidase method (GLU Glucose GOD-PAP, Roche Diagnostics, Mannheim, Germany) in a Hitachi 727 autoanalyzer. Intra- and inter-CVs were 0.9 and 1.8%, respectively. Fasting serum insulin was measured by radioimmunoassay with a commercial kit (Human Insulin Specific RIA kit, Linco Research Inc., St. Louis, MO, USA) by counting on a gamma counter (DPC Gambyt CR, Los Angeles, USA), with a lower detection limit of 2 µU/ml, intra- and inter-CVs being <1 and <7.43%, respectively. Cross-reactivity specified by the suppliers is less than 0.2% to intact human proinsulin. Serum C-reactive protein (sCRP) concentration was determined by Tina-quant CRP (Latex) high-sensitive immunoturbidimetric assay (Roche Diagnostics) in a Hitachi 917 autoanalyzer. Within-run and between-day precisions (coefficient of variation) were 0.4% and 3.4%, respectively. Adiponectin was measured by radioimmunoassay with a commercial kit (Human Adiponectin Specific RIA kit, Linco Research Inc., St. Louis, MO, USA) by counting on a gamma counter (DPC Gambyt CR, Los Angeles, USA). Intra- and inter-assay CVs were 3.73% and 8.24%, respectively.

Cigarette smokers were defined as having smoked at least 1 cigarette per day for 1 year or more. Those who reported smoking at least 1 cigarette per day during preceding year were classified as current smokers.

Each subject was assessed for the presence of MS using the National Cholesterol Education Program/Adult Treatment Panel III (NCEP/ATP III) criteria [26]: any 3 or more of the following: (1) WC>102 cm, (2) fasting TG≥150 mg/dl, (3) SBP≥130 mmHg, and/or DBP≥85 mmHg, (4) fasting HDL-C <40 mg/dl, and (5) FPG≥100 mg/dl. Insulin sensitivity was assessed with the fasting insulin, HOMA-IR using the software HOMA Calculator v.2.2.2 for Windows (www.dtu.ox.ac.uk/index.php?maindoc=/homa/) [27].

### Genotyping

Genotypes for the *TLR4* +3725*G/C* (rs11536889) polymorphism were scored blindly using allele specific PCR confronting two-pair primers analysis, as previously described [28]. The amplified DNA, visualized on a 2% agarose gel, was 256 bp for *C* allele, 184 bp for *G* allele, and 397 bp for a common band. Our lab randomly sampled 10% of the subjects for sequencing to corroborate the initial findings. Using these criteria consistency was 100%. Genotyping of SNP 11350*G/C* (rs41426344) was performed by KBioscience (http://www.kbioscience.co.uk ) using the KASPar chemistry, which is a competitive allele specific PCR SNP genotyping system using FRET quencher cassette oligos (http://www.kbioscience.co.uk/genotyping/?genotyping-chemistry.htm). Blind duplicates, plate-identifying blank wells, and Hardy-Weinberg equilibrium tests were used as quality-control tests.

### Statistical Analysis

Data on continuous variables are shown as means±SD. Categorical variables are reported as proportions (%). Deviation from the Hardy–Weinberg equilibrium was assessed using χ^2^ test. A two-tailed probability value of 0.05 was considered to be statistically significant. Odds ratios (ORs) are reported with their 95% confidence intervals (CIs). For univariate analysis, differences between groups for qualitative variables were assessed by Fisher’s exact test. One-way ANOVA was conducted for comparison of continuous variables and Levine’s test was performed to study equality of variances. Log-transformed variables were used whenever variables showed non normal distribution. Logistic regression models were used in order to adjust for age the association between variables and single nucleotide polymorphisms on TLR4 gene, for this purpose, logistic regression was used as discriminant factor. Variables that appeared to be associated in the univariate analysis were introduced into the multivariate analysis. Multiple linear regression and logistic regression analyses were used to adjust for possible confounding variables. Subjects were also grouped into tertiles to waist circumference, and differences in events among tertiles were calculated by a contingency Fisher’s exact test. In the in vitro experiments, both cell transfection and luciferase assay were performed in triplicate and analyzed using the Fisher’s exact test. Statistical significant differences were considered when p<0.05 and results were presented as their means±SD. Statistical analyses were conducted using the program for Statistical Package for the Social Sciences, version 12.0 (SPSS, Chicago, IL, USA) and Prism 4.0 (GraphPad Software, La Jolla, CA).

## Results and Discussion

### Bioinformatic Analysis of the TLR4 gene 3′UTR

Considering that variations at the 3′UTR may result in differential post-transcriptional regulation or compromised translation, bioinformatic analysis was performed in order to look for functional patterns present in this region.

**Figure 1:**
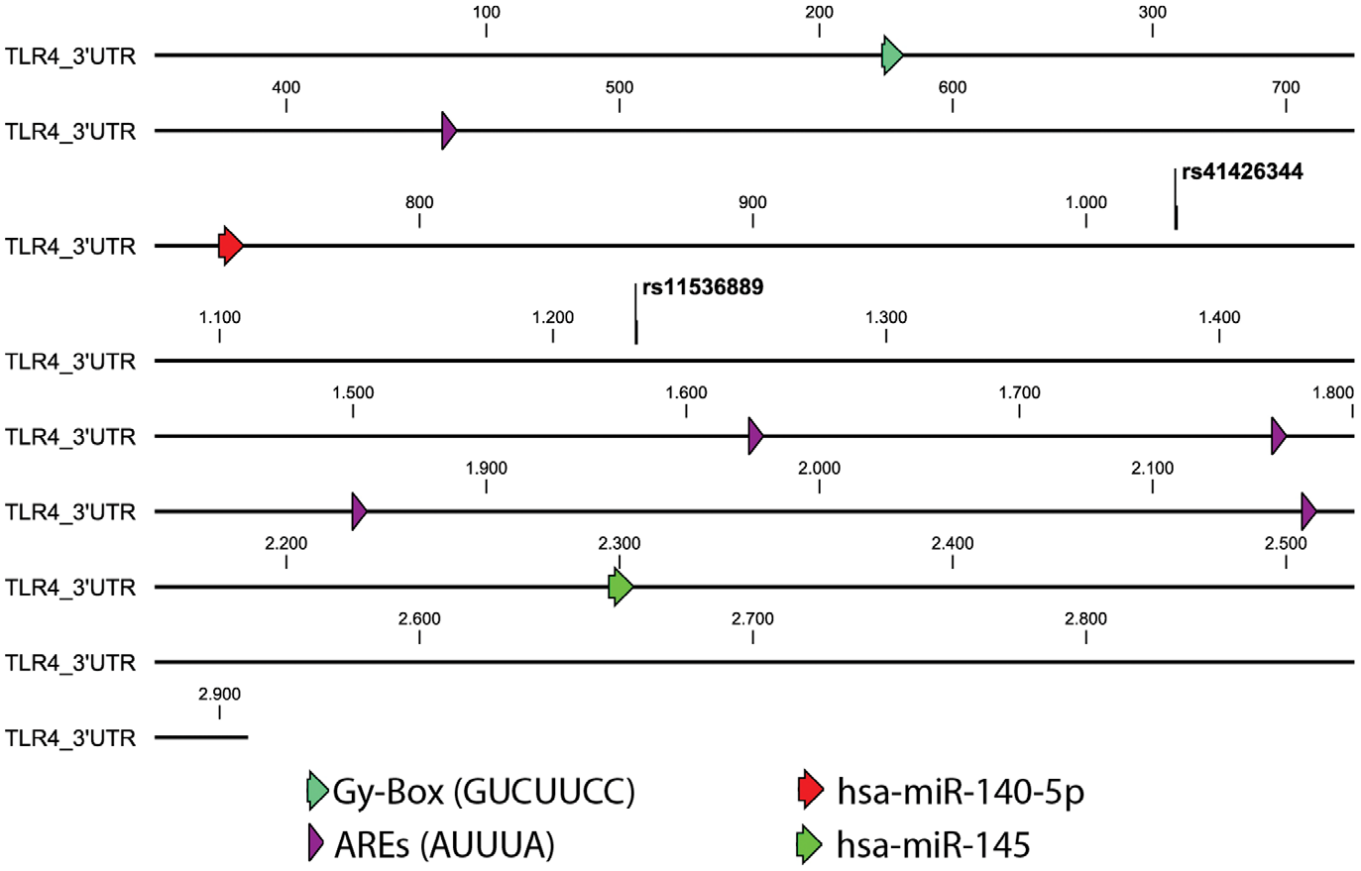
Scheme of TLR4 3′UTR, indicating the sequence variants studied and its regulatory regions.

The total length of TLR4 3′UTR determined by the University of California Santa Cruz (UCSC) genome browser was 2840 bp, with the SNP +3725 located in the middle of the 3′UTR sequence. Using the on-line UTRscan program, we found that there are five Au-rich elements (AREs) on the 3′UTR of TLR4. These are located in nucleotide positions 447–451, 1619–1623, 1776–1780, 1860–1864 and 2145–2149. The SNP +3725G/C is located between the first and the second ARE. Additionally, we found other potential regulation areas such as: a Gy-Box, at nucleotide positions 219–225, which may participate in duplex formation with complementary sequences found at 5′ ends of certain miRNAs [29]; and two putative miRNAs binding sites (hsa-miR-140-5p and hsa-miR-145) evidenced by Targetscan software (Fig. 1). Using the MULTIZ algorithm we determined that the TLR4 polymorphism +3725 G/C is located on a highly conserved region present in placental mammals. (Data not shown).

### Functional Characterization of TLR4 +3725C Variant

**Figure 2:**
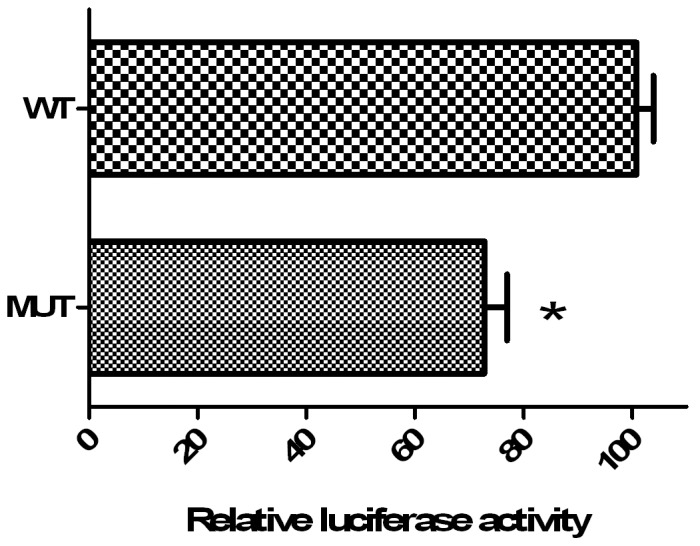
Relative Luciferase activity. Three independent assays were performed by triplicate and data represent means±SD. 24 h after transfection MUT constructs containing the +3725C variant shown lower (p = 0.0001) activity than wild-type 3′UTR construct (WT); The relative firefly Luciferase activities were normalized against β-gal.

HeLa cells were transiently transfected with the recombinant luciferase vectors (MUT or WT constructs). Twenty four hours after transfection, the β-gal-normalized luciferase activity of the MUT construct was lower than that of WT construct (p = 0.0001). In addition, the MUT construct reduced the reporter gene activity by 30% compared to that of WT construct (Fig. 2).

According to the evidence, TLR4 activation induces up-regulation of inflammatory pathways related to the induction of IR, such as IKKß/IκBα/NF-κB and JNK [30]. Interestingly TLR4 is expressed not only on innate and adaptive immune cells but also on insulin-responsive cells such as adipocytes and skeletal muscle. Muscle TLR4 over-expression has been reported in individuals with IR. Of note, other sequence variants on TLR4 gene have been associated with high risk for T2D, a condition always preceded by inflammation, MS components and IR in white populations [31]. Mice harboring loss-of-function mutations in TLR4 or CD14, a co-receptor for TLR4, have decreased body fat. Unlike many strains of caged wild-type mice, they do not become obese and the difference in body fat between mutant and wild-type mice increases with age [32].

Variations at the 3′UTR could affect several processes such as mRNA transcription efficiency, stability, post-transcriptional modification, degradation and translation. Messenger RNA containing AREs in their 3′untranslated region are particularly labile, allowing a very precise control of their temporal and spatial expression patterns. Between 5–8% of human genes code for ARE-containing mRNAs, mostly related to growth factors, oncoproteins, and cytokines. These sites bind specific proteins that regulate the mRNA stability. The presence of the +3725G/C polymorphism between the first and the second AU-rich element suggested that the SNP might influence mRNA stability, altering gene expression levels and LPS and/or another endogenous ligands response. This differential expression of TLR4 could possibly result in down-regulation of inflammatory pathways related to metabolic disorders induction.

### Characteristics of the Subjects

The average age of the subjects was 39.67±12.85 years. The clinical characteristics for all participants (966 men) are presented in Table 1. Frequency of normal weight, overweight and obesity was 23.9%, 45.3%, and 30.8%, respectively. Prevalence of current smoking and never/former smoking was 39.9% and 60.1% respectively, in the whole sample.

The prevalence of NCEP/ATP III-MS was 24.6% (n = 237). The frequency of 0, 1, 2, 3, 4 and 5 criteria for MS was 23.9% (n = 231), 27.7% (n = 268), 23.8% (n = 230), 15% (n = 145), 7.2% (n = 69), and 2.4% (n = 23), respectively.

### Genotypic Frequency of the TLR4 Polymorphisms

The DNA samples from the 966 participants were screened for the presence of two SNPs in the 3′-untranslated region (3′UTR) of the TLR4 gene. The genotype frequency of the TLR4+3725G/C polymorphism (rs11536889) was: 79.8% for G/G, 18.5% for G/C, and 1.7% for C/C, which was in Hardy–Weinberg equilibrium (χ2 = 2.97, p = 0.09). The minor allele frequency found among our population was 0.12, similar to the reported allele frequency in Caucasians (0.125 of 120 Chromosomes, HapMap project) [33]. The functional TLR4 polymorphism rs41426344 reported previously in Japanese population was not detected in our population.

### Association between TLR4 +3725G/C Polymorphism and Body Mass Index

Body mass index distribution was compared between different genotypes. The prevalence of normal weight, overweight and obesity was 53%, 35.3%, and 11.7% among individuals carrying the rs11536889 CC genotype, 25.8%; 44.1% and 30.1% for GC genotype; and 22.7%, 47.9%, and 29.4% among GG respectively (p = 0.037). The association analysis was therefore repeated taking into account different models of inheritance (dominant and recessive). The presence of the CC genotype in the TLR4 +3725G/C polymorphism under a recessive model, was associated with lean subjects, BMI less than 25 kg/m^2^ (p = 0.008), OR = 0.27 [CI 95% 0.10 to 0.70] (Table 2). The association was still significant after age adjustment (p = 0.011).

### TLR4 +3725G/C Polymorphism, Body Weight and Metabolic Disorders Stratified by Smoking Status

As hsCRP values were not normally distributed, log transformation was used. The association between log-hsCRP levels and the smoking status was studied. According to ANOVA test, smoking status was significantly associated with log-hsCRP concentration (p = 0.01) Table 1. Specifically, current smokers have higher concentrations of hsCRP (age and BMI adjusted p = 0.041).

When the sample was stratified by cigarette smoking status, the association between rs11536889 and lean status was strengthened. TLR4 +3725 G/C polymorphism was significantly associated with lean status among nonsmokers (p = 0.004), OR = 0.16 [CI 95% 0.05 to 0.56], but not among current smokers (p = 0.65) (Table 2). The association was still significant after age adjustment (p = 0.003).

**Table 2:** Fisher’s exact test analysis between rs11536889 and bmi, stratified by smoking status.

	BMI<25 kg/m^2^		BMI>25 kg/m^2^	OR	CI 95%	p	
Whole sample							
*n*	*230*		*736*				
Genotypes							
*GG+GC*	*221*		*728*	*1.0 (Ref.)*			
*CC*	*9*		*8*	*0.27*	*0.10 to 0.70*	*0.008*	
Non smokers							
*n*	*136*		*449*				
Genotypes							
*GG+GC*	*129*		*445*	*1.0 (Ref.)*			
*CC*	*7*		*4*	*0.16*	*0.05 to 0.56*	*0.004*	
Smokers							
*n*	*97*		*285*				
Genotypes							
*GG+GC*	*95*		*281*	*1.0 (Ref.)*			
*CC*	*2*		*4*	*0.67*	*0.12 to 3.76*	*0.65*	

OR, odds ratio; CI, Confidence Interval. BMI, Body Mass Index.

Taking into account these results and considering that the waist circumference (WC) is a good marker of intra abdominal adiposity, the association between CC genotype and WC was studied in nonsmokers and smokers. Among nonsmokers frequencies between CC vs. CG+GG genotypes and lower tertile of waist circumference (WC<91 cm) were significant (age adjusted OR = 0.22, p = 0.037). In this way, CC genotype carriers compared with G carriers showed significantly lower BMI (25.53±3.50 vs. 28.60±4.62 kg/m^2^; age adjusted p = 0.023), and WC (89.27±14.46 vs. 97.51±12.59 cm; age adjusted p = 0.025).

### Association between TLR4 +3725G/C Polymorphism and Adiponectin Levels

Adiponectin levels were also significantly different between CC homozygotes and the G allele carriers. It was determined an average of 14.5 ng/ml of Adiponectin in homozygous carriers of C allele, while for the GG and CG carriers the mean was 10.6 ng/ml. These differences were statistically significant (age adjusted p = 0.021). As shown before, the smoking status behaves as a confounding variable. When introducing it into the multivariate analysis, association between CC genotype and Adiponectin levels remained significant (age, BMI and smoking status adjusted p = 0.044).

### TLR4 +3725G/C Polymorphism and MS Components

The relationship between the CC genotype and the presence of MS components was studied. Since the CC genotype showed a potential relationship against development of overweight, we studied the presence of this polymorphism within individuals without any MS component vs. subjects with at least one component. Among individuals carrying the rs11536889 CC genotype, the prevalence of subjects without any MS component was 59% (n = 10) and 41% (n = 7) for subjects with at least one, and among GG and GC genotype 31% (n = 226) and 69% (n = 723), respectively (p = 0.001 age adjusted); OR = 0.166 [95% CI 0.05 to 0.49]; (age and BMI adjusted p = 0.008). These association were significant even after adjustment for confounding variables age, BMI and smoking status (p = 0.020). By studying each component individually, a non significant trend was observed consistent with former results. In particular for each MS component the following ORs were determined: WC >102 cm, OR = 0.60, p = 0.59; fasting TG ≥150 mg/dl, OR = 0.56, p = 0.38; SBP≥130 mmHg, and/or DBP≥85 mmHg, OR = 0.40 p = 0.25; fasting HDL-C <40 mg/dl, OR = 0.65 p = 0.43 and FPG ≥100 mg/dl, OR = 0.56 p = 0.47¨ age, BMI and smoking status adjusted.

### Final Discussion

We performed a functional characterization of TLR4 +3725G/C polymorphism in order to evaluate its impact on gene expression. Our findings show a substantial down regulation in gene expression when comparing luciferase activity in WT or MUT (+3725C) transfected HeLa cells. These results support the hypothesis that +3725G/C polymorphism affects mRNA stability. On the other hand this encourages further functional studies in order to unveil the mRNA regulatory mechanisms involved, and the impact of these variants in the up-regulation of inflammatory pathways related to IR.

This is the first study demonstrating significant associations between TLR4+372G/C gene polymorphism (rs11536889) and body mass index, waist circumference, Adiponectin levels and reduced risk for MS components modulated by cigarette smoking status in a general Caucasian male population.

Specifically, individuals carrying the CC genotype at the +3725 base-pair position in TLR4 gene showed lower prevalence of overweight, especially nonsmokers (never or former smokers) and higher Adiponectin levels. In addition, CC genotype was associated with lean subjects and waist circumference lower tertile only among nonsmokers. CC genotype carriers, compared with G carriers showed significantly lower BMI and WC among nonsmokers as well. Additionally, we found an association between rs11536889 and subjects without any NCEP/ATP III component for MS.

Plasmatic Adiponectin levels are known to be lower in obese than in lean subjects. Particularly a strong negative correlation between body mass index and Adiponectin serum levels has been previously shown. Our results are consistent with these observations, since Adiponectin levels were higher in CC genotype subjects.

It has also been suggested that Adiponectin has anti-inflammatory properties and acts as an endogenous modulator of obesity-related diseases through its insulin-sensitizing function. In order to study the inflammatory stage in our population, a well-known marker for systemic inflammation, CRP, was determined. A strong relationship between cigarette smoking and systemic inflammation was confirmed by numerous population-based studies: elevated levels of CRP, fibrinogen, and interleukin-6, as well as increased white blood cells counts have been reported [13]. In particular, we found that smokers show higher concentrations of CRP serum levels.

Considering that gene-environment interactions may change the effects of genetic factors by modifying environmental exposures and in particular that a low-grade systemic inflammatory response is evident in smokers, we studied the possible effects of rs11536889 in both, smokers and non smoker subjects. We found that only among non smokers, CC genotype was associated with lean subjects and waist circumference lower tertile. Taking into account that intra-abdominal adiposity correlates with waist circumference [34] portending a greater risk for diabetes and cardiovascular disease [35], the CC genotype seems to predict the development of intra-abdominal adiposity. In this way, non smokers CC genotype carriers, compared with G carriers showed significantly lower BMI and WC higher. Finally, we found association between rs11536889 and subjects without any NCEP/ATP III component for MS.

In order to explain the loss of association between rs11536889 and body weight and associated metabolic disorders in smokers, we hypothesize that cigarette might act as a confounding variable. On one hand, it is known that smokers display increased pro-inflammatory markers (e.g., TNF-α - IL-6) [36] which causes insulin resistance by interfering with insulin receptor signaling [37]. On the other hand nicotine decreases food intake and body weight by influencing the hypothalamic melanocortin system [38]. As a result of these heterogeneous interactions the predictive effect of the rs11536889 was lost in smoker subjects. Identifying a genetic profile that defines an increased risk of developing MS or cardiovascular diseases, which are well known to be extremely complex from a genetic point of view, remains one of the most difficult challenges facing research in human diseases. Although there are many associations between TLR4 polymorphisms and age-related metabolic diseases, interpretations about their protective or predictive effects are unclear and should be confirmed by further association studies. Since most current studies are underpowered to achieve such a stringent level of significance, replications are usually necessary for the confirmation of an association finding. The present study provides functional evidence of the effect of +3725G/C polymorphism in the differential TLR4 mRNA stability, in combination with an association analysis in a restricted sample population. In cross-sectional studies exposure and disease status are measured at the same time point and it may not be possible to distinguish whether the exposure preceded or followed the disease. In this way these are considered prevalence studies. However, they are useful for identifying associations and hypothesis generation. For this reason our results should be considered as an initial step and confirmed in further and independent replication cohorts.

Another important point to note is that TLR4 genetically deficient mice were reported to be an “ideal body type” when fed on regular chow, having an increased density, size and bone mineral content, as well as decreased body fat. Unlike many laboratory wild-type mice, this strain does not become obese with age. Considering that we studied a young human population (mean age of 39 years old) and that Adiponectin levels are diminished many years before the metabolic disturbances of IR are expressed, our findings are consistent with the known protective effect of Adiponectin on the development of metabolic disorders. The relationship between TLR4 and the amount and distribution of adipose tissue brings the function of TLRs in mammals much closer to that of Toll in the fly, being critical for the establishment of the body plan [39].

### Final Conclusion

we have shown for the first time a down-regulating effect of +3725G/C on TLR4 expression and an association with overweight, particularly in nonsmokers. Considering the role of TLR4 in inflammation, these findings would suggest that the presence of +3725C variant would predict a lower prevalence of chronic metabolic disorders.
